# Human Immunodeficiency Virus Risk Perception, Condom Utilization, and Associated Factors Among Youths (15–24 Years of Age) in Gashena Town, Northeast Ethiopia: Community-Based Cross-Sectional Study

**DOI:** 10.1155/arat/8874741

**Published:** 2025-02-19

**Authors:** Gebeyaw Abyie, Melaku Mekonnen, Getaw Walle

**Affiliations:** ^1^Department of Public Health, Zemen Postgraduate College, Dessie, Amhara, Ethiopia; ^2^Department of Biochemistry, College of Health Sciences, Debre Tabor University, Debre Tabor, Amhara, Ethiopia; ^3^Department of Epidemiology and Biostatistics, School of Public Health, College of Medicine and Health Sciences, Wollo University, Dessie, Amhara, Ethiopia

**Keywords:** condom utilization, Ethiopia, Gashena, risk perception, youth

## Abstract

**Background:** Globally, an estimated 1.7 million people were newly infected with human immunodeficiency virus (HIV), and approximately 37.9 million people were living with the virus. The prevalence of HIV remains high in sub-Saharan African countries, including Ethiopia. Consequently, enhancing the awareness of HIV risk is crucial for prevention efforts, as studies have shown that increased risk perception is strongly linked to condom utilization among youths.

**Objective:** This study aimed to assess HIV risk perception and condom utilization among youths and associated factors in Gashena town, Northeast Ethiopia.

**Method:** Community-based cross-sectional study involving 422 youths (15–24 years old) was conducted from June 01 to 30, 2022. Participants were selected using a systematic random sampling technique, and data analysis was conducted using Statistical Package for the Social Sciences (SPSS) Version 25 software. Logistic regression analysis was employed to determine the odds ratios for variable associations, with statistical significance set at *p* < 0.05.

**Result:** The present study revealed that 50% (95% CI: 42.7–57.3) of youths utilized condoms, while 19.4% (95% CI: 15.8–23.5) had a perception of being at risk for HIV. Factors significantly associated with both condom utilization and HIV risk perception included being 18 years old or older (AOR: 95% CI: 0.2 [0.10–0.40]), having completed primary education or higher (AOR: 95% CI: 6.23 [3.44–11.29]), and being employed (AOR: 95% CI: 1.96 [1.09–3.53]).

**Conclusion:** This study found a low prevalence of condom utilization and HIV risk perception among youths. Being 18 years old or older, having completed primary education or higher, and being employed were factors significantly linked to both condom use and HIV risk perception. Therefore, raising awareness about the implications of unprotected sexual intercourse and HIV risk perception among youths of varying ages, educational status, and occupational statuses could potentially enhance condom utilization among this demographic group.

## 1. Introduction

The human immunodeficiency virus (HIV) is the virus responsible for causing acquired immune deficiency syndrome (AIDS). Globally, it is considered the most serious public health challenge [1]. According to the 2018 global report (relatively recent report), approximately 1.7 million people were newly infected with HIV and 37.9 million were living with the virus [2]. In a single year, over 770,000 people died from HIV-related causes, and 40% of cases went undetected [3]. In response to this crisis, the United Nations General Assembly approved the “Political Declaration on HIV and AIDS in 2016: On the Fast-Track to Accelerate the Fight against HIV and to End AIDS Epidemic by 2030,” aiming to reduce new HIV infections by 75% by 2030 [4]. However, current efforts are not on track to achieve this goal, and instead, new HIV infections are on the rise. Sub-Saharan African (SSA) countries bear more than 70% of the worldwide burden of HIV infection [5]. The 2018 International AIDS Society-Lancet Commission also warned of a potential resurgence of the HIV epidemic [1, 5]. Consequently, there is an urgent need to improve HIV prevention strategies [5].

Globally, approximately 6000 new HIV infections occurred each day, with two-thirds of these new cases emerging in SSA countries, highlighting the disproportionate burden faced by different countries. Of 37.9 million individuals living with HIV, sub-Saharan countries, including Ethiopia, bore the brunt of the epidemic, accounting for around 71% of the global burden [6]. Among young females aged 15–24 years, rates of HIV infection were up to eight times higher compared to their male counterparts [7]. Therefore, recognizing individual risk factors for HIV infection is crucial for SSA countries, including Ethiopia, in their efforts to combat the spread of the virus [8]. Enhancing awareness and understanding of HIV risk is paramount for effective HIV prevention programs in SSA.

A notable research finding revealed that HIV risk perception and condom use were significantly linked among unmarried youths who engaged in multiple sexual partnerships [9]. The significance of developing accurate perceptions of HIV infection risk lies in its impact on population-wide risk perception levels. Low levels of risk perception can lead to reduced condom use, potentially contributing to higher HIV incidence rates [10, 11].

In SSA, HIV/AIDS remains a significant contributor to the overall disease burden, and various studies have highlighted substantial disparities in HIV prevalence across different countries within the region [12]. Controlling the escalating rate of new HIV infections in SSA presents numerous challenges. Factors such as population growth, sustained high incidence [13] of new HIV infections, and increased life expectancy among individuals living with HIV [14] are the primary drivers behind the growing number of people affected by HIV in SSA. However, investment in HIV prevention strategies in the region has waned in recent years, largely due to factors such as diminished economic support for healthcare [15].

In Ethiopia, approximately 715,404 people were living with HIV in 2015. This number had increased to 722,248 by 2017 [6]. HIV/AIDS continues to pose a significant public health challenge in the country, with a prevalence estimated at 1.55%, although efforts to reduce this figure are underway [16]. The issue is widespread across all Ethiopian regions, with approximately 86% of people living with HIV receiving antiretroviral therapy (ART). The prevalence of HIV infection varies from one region to another [12]. However, consistent condom utilization and perception of HIV infection risk were found to be notably lower [17, 18]. In the Amhara Regional State, HIV/AIDS presents as a diverse public health concern, influenced by factors such as location, time, gender, and age group. From 2015 to 2018, the overall incidence rate of HIV in the region was 6.9 per 1000 individuals [19].

It is crucial to implement international and national strategies aimed at reducing new HIV infection risk and increasing its preventive approaches [11]. However, there was no consistent report on HIV risk perception and condom utilization. There is also a lack of studies on HIV risk perception and condom utilization among youth in the Wollo population. Thus, this study aimed to assess HIV risk perception and condom utilization among youth and to identify factors associated with their perception of HIV risks and condom utilization.

## 2. Methods and Materials

### 2.1. Study Area and Period

This study was conducted from June 01 to 30, 2022, in Gashena town, an administrative town located in the North Wollo administration zone of the Amhara National Regional State, Northern Ethiopia. The town is found 565, 247, and 111 km far from Addis Ababa, Bahir Dar, and Woldia towns, respectively. The town is divided into four kebeles (the smallest administrative units), and it has a total population of 46,888 (22,174 males and 24,714 females). In the town, there are a total of 16,134 youths (7011 and 9123 are males and females, respectively).

### 2.2. Study Design

A community-based cross-sectional study was designed to examine HIV risk perception and condom utilization among the youth residing in Gashena town, North Wollo administration zone of the Amhara Regional State, Northern Ethiopia. The sample consisted of youth residing in the town throughout the study duration.

### 2.3. Eligibility Criteria

The study included all youths who have been living in Gashena town for at least 12 months to identify the residents of the town by considering the kebele administrates can accomplish all the tasks to deliver identification card of individuals within a year. Youths who were unable to hear and communicate verbally and those critically and mentally ill were excluded from the study.

### 2.4. Sample Size Determination and Sampling Techniques

#### 2.4.1. Sample Size Determination

The sample size was calculated by using a single population proportion formula:

(1) $$\begin{array}{c} \frac{n={\left(Z_{1-ά/2}\right)}^{2}P\left(1-P\right)}{d^{2}}, \end{array}$$

where *n* is the minimum sample size required and Z_1-*α*/2_ is the standard normal variable at (1 − *α*) % confidence level and at *α* level of significance. A 95% confidence level (1.96) and 5% margin of error (*d* = 0.05) are used. Based on the study conducted at Debre Birhan town, the prevalence of youth perceiving themselves as being at risk of acquiring HIV infection is only about 4.5% (*p* = 0.045) in the Amhara region [20]. Therefore, to find the appropriate sample size, the current study used a prevalence of 50% (*p* = 0.5), and by adding a 10% nonresponse rate, the sample size (*n*) becomes 422. The prevalence of consistent condom use among youth is 44.9% (*p* = 0.449) in the Amhara region [20]. Thus, using a prevalence of 44.9% (*p* = 0.449) and adding a nonresponse rate of 10%, the sample size (*n*) becomes 418. Then, after calculating separately, this study took a larger sample size (422).

#### 2.4.2. Sampling Techniques

There are 4 kebeles in Gashena town, and all of these kebeles were included in the present study. The numbers of households from each kebeles were determined based on their population size using a systematic random sampling technique with independent sampling intervals (*k*th value) applied to each kebele. Here, the sample size was also allocated proportionally according to the number of households in each kebele. Only one individual youth in each household was selected and included in this study. If more than one eligible youth were found in a single household, we selected only one of them by the lottery method.

### 2.5. Study Variables

HIV risk perception and condom utilization were dependent variables whereas sociodemographic characteristics (age, sex, marital status, educational status, and occupational status), sexual behaviors, peer pressure, alcohol consumption, cigarette smoking, and chat chewing were independent variables.

### 2.6. Operational Definitions

Operational definitions are as follows:

• Youths: Youths who are in the age group of 15–24 years [21].
• HIV Risk Perception: Whether youths perceive themselves as being susceptible to HIV infection [22].
• Condom Utilization: Whether the study participants always use condoms regularly and consistently during their sexual intercourse [23].
• Knowledge of Youth Toward Risks of Sexual Activities: Youths who answered at least 7 of the 9 (≥ 70%) knowledge assessing questions were considered as having good knowledge when analyzed [23].
• Attitude of Youth Toward Risks of Sexual Activities: Youths who answered at least 6 of the 8 (≥ 70%) attitude assessing questions were considered as having favorable attitude during analysis [23].

### 2.7. Data Collection Procedures

After a brief explanation was given to participants, formal written consent was taken for their willingness to participate in the study. Then, the responses of participants were taken by using structured and interviewer-administered questionnaires. Two data collectors (clinical nurses) were recruited from the local community since they would understand the cultural activities of the community and could approach youth in harmony with norms to get the necessary responses. Male and female data collectors were assigned for male and female participants, respectively, due to the sensitivity of issues so that the youth would answer and give appropriate responses without fear. The data collectors were supervised by researchers.

The knowledge section comprised 9 items, with correct answers scored 1 point and incorrect answers scored 0 points, resulting in a hypothetical score range of 0 to 9 points, while the attitude section of the questionnaire included 8 items with correct answers scored 1 point and incorrect answers scored 0 points, resulting in a hypothetical score range of 0 to 8 points.

### 2.8. Data Quality Assurance

The questionnaire was meticulously designed by the researchers by reviewing various peer-reviewed published articles and tailored to align with specific objectives of the current study [17, 18, 20]. Primarily, the questionnaire was prepared in English, and then, it was translated into the local Amharic language by the research team to facilitate effective communication. Finally, it was translated back to English to be used. The researchers are native speakers of the local Amharic language so they properly applied the forward and backward translation process.

Health professional (nurses) data collectors were selected based on their experience in data collection, and they were well informed on the objectives of the study, interview techniques, and ethical considerations. A pilot study was carried out on 5% of study participants in the study area to pretest and assure data validity. The data obtained in the pilot study were analyzed by applying Cronbach alpha, the commonly used statistical technique to assess questionnaire validity. The Cronbach alpha values (coefficients) of knowledge and attitude assessing questions were 0.94 and 0.91, respectively, indicating excellent reliability. The researchers also conducted daily revisions of the collected data to check for errors, legibility, completeness, and consistency before entering it into the database for statistical analysis. Any mistakes or ambiguities were addressed on the spot.

### 2.9. Data Processing and Statistical Analysis

The research team verified completeness, coded and entered the data into Epi Data Version 3.1, which was then exported to Statistical Package for the Social Sciences (SPSS) Version 25 for analysis. The researchers utilized descriptive statistics, including frequencies, means, and proportions. Variables with a *p* value < 0.25 in bivariable logistic regression were involved in the multivariable model to identify independent associations. Adjusted odds ratios (AOR) with 95% confidence intervals (CI) were reported, with a significance level set at *p* < 0.05.

### 2.10. Ethical Considerations

The current study was conducted following the Declaration of Helsinki. Ethical approval was obtained from the Zemen Post Graduate College Coordinating Program Ethical Review Committee through a formal letter having ethical approval code “ZPGC/00325/14” written on “14/07/14 E.C.” The objectives of the study were explained to the officials of Gashena town and each study participant. Written informed consent was signed by each study participant prior to data collection. The youths were also informed that participating in the study was voluntary and they had a right in order not to participate. The youths were assured that confidentiality of the data taken would be kept safe and only be used for the research purpose. The informed consent was obtained from their parents or caregivers if the participants were under 18 years of age.

## 3. Results

### 3.1. Sociodemographic Characteristics of Youths

About 37% and 63% of the youths were found under the age group of less than 18 years old and 18 years old and above, respectively. About 53.6% of youths were females, and 65.9% were orthodox religion followers. In terms of ethnicity, 72.7% of them were found to be Amhara. About 88.6% of the youths were found to be single in marital status. The study also showed that 79.1% of the youths had primary educational status and above (Table 1).

### 3.2. Behavioral Characteristics of Youths

Of the total youths, 20.6%, 24.2%, and 22.3% had a history of cigarette smoking, alcohol consumption, and chat chewing, respectively. About 27% of them were also found to be influenced by peer pressure influence (Table 2).

### 3.3. Knowledge and Attitude of Youths Toward Condom Utilization

Around 18.2% and 37% of the study participants had poor knowledge about condom utilization and unfavorable attitude toward condom utilization, respectively (Table 3).

### 3.4. Sexual Experience of the Youths

Approximately 45% of youths had previous sexual experiences, with 63.2% of them initiating sexual activity before the age of 18 years. Around 37.9% of youths began their sexual intercourse with a steady partner. Of those who had engaged in sexual intercourse, 44.7% cited peer pressure as a contributing factor to their first sexual encounter. Notably, only 50% (95% CI: 42.7–57.3) of youths used condoms during their first sexual experience, while approximately 34.7% never used condoms (Table 4).

### 3.5. HIV Risk Perception by the Youths

About 19.4% (95% CI: 15.8–23.5) of youths perceived themselves as being at risk of HIV/AIDS infection. Of the youths who perceived themselves as being at risk of HIV/AIDS infection, 54.9% of them listed performing sex without condom as a reason for their perception to be at risk for HIV infection (Table 5).

### 3.6. Factors Associated With Condom Utilization

Age, occupational status, attitude toward condom use, age at first sexual intercourse, and first sexual experience with a casual friend were variables with *p* values less than 0.25 in bivariable analysis and included in a multivariable logistic regression model to determine independently significant predictor variables using AOR with a 95% CI. The multivariable logistic regression revealed that youth aged 18 and above were more likely to use condoms compared to their counterparts (AOR: 0.16 [95% CI: 0.03–0.78]). In addition, youths with favorable attitude toward condom use were more inclined to utilize condoms compared to those with unfavorable attitude (AOR: 0.01 [95% CI: 0.001–0.03]). Furthermore, individuals who were 18 years or older at their first sexual encounter were more likely to use condoms compared to their counterparts (AOR: 0.08 [95% CI: 0.02–0.37]). Besides, youths who engaged in their first sexual intercourse with a casual sexual partner were more likely to use condoms compared to their counterparts (AOR: 0.003 [95% CI: 0.000–0.024]) (Table 6).

### 3.7. Factors Associated With HIV Risk Perception

Age, educational status, and occupational status were identified as variables with *p* values less than 0.25 in bivariable analysis and subsequently included in a multivariable logistic regression model to determine independently significant predictor variables using AOR with a 95% CI. The multivariable logistic regression analysis revealed that the aforementioned variables were significantly associated with HIV risk perception. Young people in the age group of 18 years and above were more likely to perceive themselves as at risk for HIV infection compared to their counterparts (AOR: 0.2 [95% CI: 0.10–0.40]). Similarly, youths who had completed primary education or higher were more inclined to perceive themselves as at risk for HIV infection compared to illiterates (AOR: 6.23 [95% CI: 3.44–11.29]). In addition, jobless youths were more likely to perceive themselves as at risk for HIV infection as compared to employed youths (AOR: 1.96 [95% CI: 1.09–3.53]) (Table 7).

## 4. Discussion

This study aimed to assess the prevalence and factors associated with condom use and HIV risk perception among youths. The overall rates of condom utilization and HIV risk perception among youths were found to be 50% (95% CI: 42.7–57.3) and 19.4% (95% CI: 15.8–23.5), respectively. Despite condoms being a key preventive measure for approximately 98% of sexually transmitted diseases, including HIV/AIDS [4], the observed utilization rate is considered low. Given that all sexually active youths are at higher risk of HIV infection, consistent and regular condom use is crucial. These findings align with previous global studies indicating lower condom utilization rates among students engaging in risky sexual behaviors [24]. Similarly, research in Taiwan revealed low condom usage during initial sexual encounters, suggesting that many sexually active adolescents may not be practicing safe sex. A study in Egypt also reported a low prevalence of condom use in sexual relationships (45.0%) [25]. However, the result of this study contrasts with a 2019 US Youth Risk Behavior Survey, which showed only 9% of sexually active high school students using condoms for HIV/AIDS prevention [24]. This discrepancy might be due to the inclusion of youths in this study who were not limited to school settings but also engaged in various activities, that could potentially increase their HIV risk. This discrepancy suggests that despite the current lower rates, condom use among youths in Gashena town is showing signs of improvement and approaching levels seen in developed countries such as the United States among school-going youth.

The current study uncovered several significant factors associated with condom utilization and HIV/AIDS risk perception among young individuals. Specifically, factors such as being 18 years old or above, having a favorable attitude toward condom use, engaging in first sexual intercourse at the age of 18 or older, and having the first sexual experience with a casual friend were found to be statistically significant predictors of condom utilization among youths. Similarly, being 18 years old or above, having completed primary education or higher, and being employed were also identified as statistically significant factors associated with the level of HIV/AIDS risk perception.

Age emerged as a significant predictor of condom utilization, with individuals aged 18 and above exhibiting higher rates of condom use compared to their younger counterparts. This observation is corroborated by research conducted among individuals living with HIV/AIDS in Egypt and a study in Addis Ababa, Ethiopia [23, 25]. This disparity in condom use may be attributed to limited access to information on reproductive health issues and the immediate and long-term consequences of sexual violence among youths and the broader community. Furthermore, age was also found to be a significant factor in HIV risk perception, with individuals aged 18 and above demonstrating greater awareness of HIV risk compared to younger individuals. This aligns with findings from a previous study conducted in Ethiopia.^38^

Likewise, the attitude of young individuals toward condom use emerged as a significant factor associated with condom utilization. This study revealed that young people who held a favorable attitude toward condom utilization were approximately six times more likely to use condoms compared to those who had an unfavorable attitude toward condom use. This disparity may be attributed to the fact that having a favorable attitude toward something increases the likelihood of engaging in that particular behavior. These findings are supported by research conducted among people living with HIV/AIDS in Egypt and a study carried out in Addis Ababa, Ethiopia [23, 25].

Other factors associated with condom use among young people included the age at which they first had sexual intercourse and the identity of their sexual partner during their first sexual encounter. The current study revealed that young individuals aged 18 and above and those who engaged in their first sexual experience with casual partners were more likely to use condoms compared to their counterparts. This could be attributed to the fact that older youths may feel more confident and empowered to make decisions about condom use. Similarly, young people who had casual partners may be more inclined to use condoms as a means of self-protection. These findings are supported by previous studies conducted in Malaysia, Ghana, and Ethiopia, which also demonstrated a statistically significant association between condom use and the type of sexual partner [23, 25–27].

### 4.1. Strengths and Limitations of the Study

The study's key strength lies in its originality, as it provides insight into the assessment of HIV risk perception and condom utilization among youths and associated factors in Gashena town, Northern Ethiopia. This serves as a foundational reference for future in-depth research. There was the challenge of obtaining responses to sexuality-related questions from youths in face-to-face interviews. As a result, there may have been a predisposition to social desirability bias, despite efforts to collect data anonymously by arranging male and female data collectors for male and female youths, respectively, was carried out. In addition, the study's cross-sectional design limited its ability to determine causal relationships.

## 5. Conclusion

The prevalence of condom use and perception of HIV risk among youths was found to be low, highlighting the need for consistent condom use during sexual encounters with casual partners. Being 18 years or older, having a favorable attitude toward condom use, initiating sexual activity at or after the age of 18, and engaging in first sexual intercourse with casual partners were factors significantly associated with increased condom utilization among youths. In addition, being 18 years or older, having completed primary education or higher, and being employed were factors significantly linked to greater awareness of HIV risk.

Given these findings, there is an urgent need for widespread awareness campaigns on the importance of condom use and understanding HIV risk among youths. It is crucial for health sectors at various administrative levels, including district, zonal, regional, and federal authorities, as well as other stakeholders, to prioritize efforts to increase condom utilization and enhance HIV risk perception among young individuals. Regular health promotion initiatives focusing on sexual and reproductive health issues should be implemented to address these concerns effectively.

## Figures and Tables

**Table 1:** Sociodemographic features of youths in Gashena town, Amhara region, Ethiopia, 2022.

Variables		Frequency	Percent
Age	< 18	156	37
	≥ 18	266	63

Sex	Male	196	46.4
	Female	226	53.6

Religion	Orthodox	278	65.9
	Muslim	92	21.8
	Others	52	12.3

Ethnicity	Amhara	307	72.7
	Others	115	27.3

Educational status	Illiterate	88	20.9
	Primary education and above	334	79.1

Marital status	Married	48	11.4
	Single	374	88.6

Occupational status	Government/private employee	105	24.9
	Jobless	317	75.1

Income in Ethiopian Birr	< 5000	366	86.7
	≥ 5000	56	13.3

*Note:* All variables are continuous so that they are presented as number and percentage. Other refers to protestants and Catholics for religion and Oromo, Gurage, and Wolaita for ethnicity.

**Table 2:** Behavioral characteristics of youths in Gashena town, Amhara region, Ethiopia, 2022.

Variables		Frequency	Percent
Cigarette smoking	Yes	87	20.6
	No	335	79.4

Alcohol consumption	Yes	102	24.2
	No	320	75.8

Chat chewing	Yes	94	22.3
	No	328	77.7

Presence of peer influence	Yes	114	27
	No	308	73

*Note:* All variables are continuous so that they are presented as number and percentage.

**Table 3:** Knowledge and attitude among youths in Gashena town, Amhara region, Ethiopia, 2022.

Variables		Frequency	Percent
Knowledge	Good	345	81.8
	Poor	77	18.2

Attitude	Favorable	266	63
	Unfavorable	156	37

*Note:* All variables are continuous so that they are presented as number and percentage.

**Table 4:** Sexual experiences among youths in Gashena town, Amhara region, Ethiopia, 2022.

Variables		Frequency	Percent
Ever had sex	Yes	190	45
	No	232	55

Age at 1^st^ sex (years)	< 18	120	63.2
	≥ 18	70	36.8

You made your first sex with	Steady friend/wife/husband	72	37.9
	Causal friend	118	62.1

Reason for sex	Peer pressure	85	44.7
	Self-interest	105	55.3

Condom use during your 1^st^ sex	Yes	95	50
	No	95	50

Frequency of condom use	Always	35	18.4
	Mostly	40	21.1
	Sometimes	49	25.8
	Never	66	34.7

Reason to use a condom	To prevent HIV	86	68.3
	To prevent unwanted pregnancy	40	31.7

Reason for infrequent use of a condom	Lack of availability	23	14.8
	Expensiveness	43	27.7
	Being ashamed	4	2.6
	Prohibited by religion	28	18.1
	Want child	31	20
	I do not know how to use	26	16.8

*Note:* All variables are continuous so that they are presented as number and percentage.

**Table 5:** Assessment of HIV risk perception among youths in Gashena town, Amhara region, Ethiopia, 2022.

Variables		Frequency	Percent
Risk perception	Yes	82	19.4
	No	340	80.6

Reason for risk perception	Sex without condom	45	54.9
	Sex with a commercial sex worker	11	13.4
	Sex during alcohol intoxication	12	14.6
	Sex during chat chewing	14	17.1

*Note:* All variables are continuous so that they are presented as number and percentage.

**Table 6:** Factors associated with condom utilization among youths in Gashena town, Amhara region, Ethiopia, 2022.

Variables		Condom utilization		COR (95% CI)	AOR (95% CI)	*p* value
		Yes	No			
Age (years)	< 18⁣^∗^	16	32	1	1	**0.023**
	≥ 18⁣^∗∗^	79	63	0.40 (0.20–0.79)	0.16 (0.03–0.78)	

Attitude	Favorable⁣^∗∗^	91	37	1	1	**≤ 0.001**
	Unfavorable⁣^∗^	4	58	0.03 (0.01–0.08)	0.01 (0.001–0.03)	

Age at first sex (years)	< 18⁣^∗^	49	71	1	1	**≤ 0.001**
	≥ 18⁣^∗∗^	46	24	0.36 (0.20–0.67)	0.08 (0.02–0.37)	

You made your first sex with	Steady friend⁣^∗^	11	61	1	1	**≤ 0.001**
	Causal friend⁣^∗∗^	84	34	13.7 (6.44–29.17)	0.003 (0.000–0.024)	

*Note:* Those categories having variables marked by single strikes (⁣^∗^) are reference categories while those categories having variables marked by double strikes (⁣^∗∗^) contain significant variables to which the COR and AOR was carried out. Bold values represent the significant values.

**Table 7:** Factors associated with HIV risk perception among youths in Gashena town, Amhara region, Ethiopia, 2022.

Variables		Risk perception		COR (95% CI)	AOR (95% CI)	*p* value
		Yes	No			
Age category (years)	< 18⁣^∗^	12	144	1	1	**≤ 0.001**
	≥ 18⁣^∗∗^	70	196	0.23 (0.12–0.45)	0.2 (0.10–0.40)	

Educational status	Illiterate⁣^∗^	36	52	1	1	**≤ 0.001**
	≥ Primary education⁣^∗∗^	46	288	4.33 (2.56–7.34)	6.23 (3.44–11.29)	

Occupational status	Govt./private employee⁣^∗^	28	77	1	1	**0.025**
	Jobless⁣^∗∗^	54	263	0.57 (0.34–0.95)	1.96 (1.09–3.53)	

*Note:* Categories having variables marked by single strikes (⁣^∗^) are reference categories while those categories having variables marked by double strikes (⁣^∗∗^) contain significant variables to which the COR and AOR was carried out. Bold values represent the significant values.

## Data Availability

The datasets used and/or analyzed during the present study are available from the corresponding author upon reasonable request.

## Nomenclature

AIDS: Acquired immune deficiency syndrome
HIV: Human immunodeficiency virus
SSA: Sub-Saharan Africa
